# Attention biases in female survivors of chronic interpersonal violence: relationship to trauma-related symptoms and physiology

**DOI:** 10.3402/ejpt.v4i0.19135

**Published:** 2013-03-04

**Authors:** Jonathan DePierro, Wendy D'Andrea, Nnamdi Pole

**Affiliations:** 1 Psychology Department, The New School for Social Research, New York, NY, USA; 2 Psychology Department, Smith College, Northampton, MA, USA

**Keywords:** Posttraumatic stress disorder, interpersonal violence, psychophysiology, attention bias

## Abstract

**Background:**

Exposure to chronic interpersonal violence (IPV) has been associated with psychiatric impairment; however, few studies have investigated attention processes and psychophysiology in this population.

**Objective:**

We investigated self-report and physiological correlates of attention biases in 27 IPV-exposed women.

**Method:**

Participants completed self-report measures of trauma history, posttraumatic stress disorder (PTSD) symptoms, and dissociation; were monitored physiologically during baseline; and responded to an emotional dot probe task.

**Results:**

Participants showed bias away from positive and anxiety words, and toward IPV words. Lower baseline respiratory sinus arrhythmia (RSA) and higher skin conductance levels were associated with bias away from anxiety cues. Greater total PTSD symptoms were associated with bias toward IPV cues, and greater PTSD intrusion and avoidance symptoms were associated with lower RSA. Individuals exposed to more types of trauma had lower heart rates.

**Conclusions:**

These data extend the research on emotion–cognition interactions in PTSD and other anxiety disorders to chronic IPV survivors, in part confirming avoidance and intrusion symptom and attention bias relations found in studies. The present work also draws attention to a group that tends to experience a range of severe symptoms associated with apparent blunting in autonomic activity, and suggests that self-report may not be sensitive to physiological and attention alterations in chronic IPV samples.

Interpersonal violence (IPV) is more likely than other forms of trauma to lead to posttraumatic stress disorder (PTSD) (Scott, 2007). IPV survivors also report difficulties with attention and emotion regulation (van der Kolk, Roth, Pelcovitz, Sunday, & Spinazzola, 2005), have more complex and severe symptoms than survivors of non-IPV traumas (Ford, Stockton, Kaltman, & Green, 2006), and evidence both too much and too little autonomic activity (Corrigan, Fisher, & Nutt, 2011). However, few studies have examined inter-relationships among PTSD, attention, and physiology in IPV survivors. Concurrently, the literature on attention in PTSD (e.g. Bryant & Harvey, 1997; Elsesser, Sartory, & Tackenberg, 2004) has yielded mixed findings, perhaps due to variability in PTSD symptom and trauma exposure. The present study will attempt to fill gaps in the literature by examining relations among attention bias, self-report, and physiology in female survivors of IPV and may help disentangle inter-related attentional and physiological mechanisms that could undergird PTSD symptoms.

## Attention biases for threat in PTSD

The vigilance–avoidance hypothesis (Mogg, Bradley, Miles, & Dixon, 2004) of anxiety disorders suggests that affected individuals show both vigilance toward (selective attention or attention bias toward) and avoidance of (attention bias away from) anxiety reminders. Vigilance–avoidance has examined using the dot probe task (MacLeod, Mathews, & Tata, 1986). On this task, faster identification speed for a probe (e.g. a cross) presented in spaces previously occupied by threat cues is interpreted as increased threat vigilance (MacLeod et al., 1986) or difficulty disengaging (Koster, Crombez, Verschuere, & De Houwer, 2004), and hastened reaction time when a probe presented in spaces opposite cues is interpreted as avoidance.

Bryant and Harvey (1997) found bias toward mildly threatening verbal stimuli on the dot probe for PTSD+ motor vehicle accident survivors compared to trauma-exposed but PTSD− comparisons. Felmingham, Rennie, Manor, and Bryant (2011) found that PTSD+ individuals showed increased fixations on threat cues, supporting for the vigilance portion of the vigilance–avoidance hypothesis. Dalgleish, Moradi, Taghavi, Neshat-Doost, and Yule (2001) found bias away from threat on a verbal dot probe in a sample of children with PTSD related to road traffic accidents or exposure to violence, and Wald et al. (2011) used bias away from threat to predict later PTSD in adults exposed to rocket attacks. In contrast, Elsesser et al. (2004) found no group difference in bias toward threat on the dot probe comparing those with chronic PTSD to controls, and Fani, Bradley-Davino, Ressler, and McClure-Tone (2010) found no associations between trauma-related symptoms (or childhood trauma exposure) and threat bias with this task in adult survivors of childhood maltreatment. Overall findings are mixed, with studies reporting that PTSD is related to bias to threat, bias away from threat, and no bias.

One factor that might be associated with these mixed findings is that PTSD symptoms are themselves heterogeneous. PTSD symptoms have been conceptualized as extreme approach or withdrawal states (Stein & Paulus, 2009) that accompany changes in physiology (Pole, 2007), and this variability may impact on attention. Some studies have attempted to clarify findings by examining symptom clusters. Elsesser et al. (2004) found that intrusion symptoms were related to biases away from threat on the dot probe, and Teachman, Smith-Janik, and Saporito (2007) showed that increased avoidance symptoms in anxious adults were associated with greater threat avoidance on the Stroop task.

Further heterogeneity in symptom profiles may be attributed to PTSD's associated features. IPV survivors may report symptoms of dissociation (Cloitre et al., 2009), the disintegration of the normally integrated functions of consciousness, memory, identity, and perception (American Psychiatric Association [APA], 2000). Empirical work relates dissociation to alterations in attention, though studies using the dot probe are scarce. Dorahy, Middleton, and Irwin (2005) demonstrated slowed response to negative words on a flanker task in individuals with dissociative identity disorder, and Mueller-Pfeiffer et al. (2010) found that slowed response to negative stimuli on the Stroop task was related to task-related depersonalization in PTSD+ individuals. As dissociation is often more severe in individuals with chronic trauma histories (van der Hart, Nijenhuis, & Steele, 2005), dissociation may be related to attention in this group.

## Integrating physiology and attention in trauma survivors

How physiology relates to attention in chronically traumatized individuals has been rarely studied, though data on attention and physiology in healthy and acutely traumatized samples suggest this area merits study. Early research on cognition–physiology interactions established that autonomic arousal impacts attention (Yerkes & Dodson, 1908). This work suggests that both too little and too much arousal negatively impact cognition; instead, an optimal degree of arousal is necessary for engaging in attention tasks. Pharmacological studies demonstrate that either blocking or enhancing autonomic arousal impacts attention (Thayer, Hansen, Saus-Rose, & Johnsen, 2009); other research has shown that engaging in attention-focused tasks affects physiology (Siegle, Granholm, Ingram, & Matt, 2001).

The autonomic nervous system (ANS) is comprised of sympathetic (SNS) and parasympathetic (PNS) branches (Andreassi, 2007). SNS activity corresponds to both stress and cognitive engagement. Sweat gland activity, called skin conductance level (SCL), provides a measure of SNS activity (Andreassi, 2007). For example, SCL increases to task-relevant stimuli in selective attention paradigms (e.g. Filion, Dawson, Schell, & Hazlett, 1991), and during exposure to masked phobic stimuli in phobic individuals (Ohman & Soares, 1994). SNS activity and reactivity tend to be higher in PTSD+ individuals (Pole, 2007). In addition, elevated SNS activity among trauma-exposed individuals may mediate the relationship between trauma exposure and attention. Elsesser et al. (2004) found that increased heart rate while viewing trauma-relevant pictures was correlated with increased bias toward trauma material on a later dot probe task in PTSD+ individuals, and Felmingham et al.'s (2011) PTSD group showed increased skin conductance reactivity associated with threat fixations.

Like the SNS, the PNS integrates with both emotion regulation (Porges, 2007) and cognitive processes including attention (for review, see Thayer et al., 2009). Vagal activity, indexed by respiratory sinus arrhythmia (RSA), provides a measure of PNS activity (Berntson, Cacioppo, & Quigley, 1993). Increased baseline PNS activity is linked to better executive control and lower anxiety (e.g. Hansen, Johnsen, & Thayer, 2003; Thayer, Friedman, & Borkovec, 1996). Individuals with high trait anxiety who have low baseline PNS activity show difficulty disengaging from threat (Cocia, Uscatescu, & Rusu, 2012). PNS activity appears to be blunted in individuals with trauma exposure (Hopper, Spinazzola, Simpson, & van der Kolk, 2006; Sack, Hopper, & Lamprecht, 2004). However, these latter studies have not explicitly included individuals with multiple early lifetime traumas or report on the relationship between chronic trauma and physiology. Further, hyperarousal findings in PTSD may reflect either SNS excess or PNS depletion (e.g. Sack et al., 2004); therefore, measures which selectively reflect SNS and PNS activation may clarify mechanisms of hyperarousal.

While individuals with complex trauma exposure might be expected to exhibit high physiological arousal (especially in the SNS), recent findings have suggested blunted activity is also typical (Kraus et al., 2009; McTeague et al., 2010). Earlier work by Griffin, Resick, and Mechanic (1997) demonstrated that rape survivors high on peritraumatic dissociation had blunted skin conductance activity during script driven imagery. Low states of arousal are likely to bear on attention, including attention to threat (Corrigan et al., 2011). Though prior work provides evidence for a possible link between ANS activity, attention, and symptom development, research attending to PTSD symptom clusters and both ANS branches may clarify attention biases toward and away from threat reminders.

## The present study

The present study, involving female survivors of chronic IPV, extends the existing literature on the relations between trauma exposure and attention by including an examination of symptom cluster and trauma history, and PNS and SNS activity. We addressed the following questions: (a) Is attention to emotional stimuli related to PTSD symptoms and dissociation, or to trauma variables (age of first exposure and range of exposure)? (b) Is attention to emotional stimuli related to baseline physiology (e.g., heart rate [HR], RSA, and SCL)? We examined age of onset of trauma and exposure to multiple forms of IPV because they appear to link IPV itself to psychological distress (e.g. Breslau et al., 1998; Ford et al., 2006), and relations among these history variables to attention and physiology are unclear.

## Method

### Participants

Participants consisted of 27 adult treatment-seeking women exposed to IPV (e.g., rape, childhood physical or sexual abuse, and domestic violence) who were recruited into a larger study (D'Andea & Pole, 2012). Physiological data were available for 23 of the 27 participants. The mean age for participants was 37.5 (*SD*=13.9). The sample was 69% Euro-American, 17% Native American, 9% African American, and 4% Asian American. Eighty-two percent attended or completed college; 87% identified as heterosexual and 13% as lesbian or bisexual; and 56% were using psychotropic medications. Eighty-three percent of the sample was right handed.

### Materials

#### The dissociative experiences scale

Dissociative Experiences Scale (DES; Carlson & Putnam, 1993) is a 28-item self-report measure of dissociative experiences (depersonalization, derealization, absorption, and amnesia). Participants indicated the frequency of each symptom in the last month (i.e., from 0 to 100% of the time). A mean score of 30 raises the likelihood of meeting criteria for dissociative disorders (Carlson & Putnam, 1993), and the same cut-off on the DES pathological dissociation items serves as a more conservative measure (Waller & Ross, 1997). The DES has been validated against structured interview (for meta-analysis, see van IJzendoorn, & Schuengel, 1996). Test–retest reliability ranges from *r*=0.78–0.96 (Dubester & Braun, 1995). Internal consistency was high in the present study (alpha=0.96). This measure does not assess fragmentation, an important component of dissociation.

### The Posttraumatic Stress Disorder Checklist

Posttraumatic Stress Disorder Checklist (PCL; Weathers, Litz, Herman, Huska, & Keane, 1993) is a self-report measure comprising DSM-IV PTSD criteria B through D (re-experiencing/intrusion, avoidance/numbing and hyperarousal symptoms). Participants rated symptoms over the past month on a 5-point Likert scale. For diagnostic purposes only (and not for analyses), we modified the PCL to include both frequency *and* intensity scores to be consistent with the Clinician-Administered PTSD Scale (CAPS; Blake et al., 1995), and then applied the algorithm for PTSD diagnosis established for the CAPS (Weathers, Ruscio, & Keane, 1999) to estimate PTSD status. Test–retest reliability is high (*r*=0.92; Ruggiero, Del Ben, Scotti, & Rabalais, 2003). Internal consistency for the three subscales used here varied from weak to good (alphas=0.67–0.82).

### The Trauma History Questionnaire

Trauma History Questionnaire (THQ; Green, 1996) assessed lifetime exposure to traumatic events (e.g., accidents, sexual assaults) and was modified to also assess age of first and last experience of each event. The THQ was reduced to three variables: (1) age of first and (2) latest traumatic event, and (3) number of different types of events (e.g., sexual assault plus physical assault=2).

### Dot probe task

We used a dot probe task abbreviated relative to other versions (e.g. Macleod et al., 1986; Mogg et al., 1992) to diminish participant burden, and the task was presented on a computer using the STIM Auditory/Visual Presentation and Laboratory Control program (James Long Company [JCL], Caroga Lake, NY). There were two practice and 53 unique experimental trials pairing target (11 neutral, 10 positive, 13 anxiety, and 20 IPV words) and neutral words that shared two-letter stems. Words were adapted from previous studies (Bradley & Lang, 1999; Foa, Feske, Murdock, Kozak, & McCarthy 1991; Paunovic, Lundh, & Ost, 2002). IPV words were oversampled to capture varying participant experiences. Trial order was randomized and the same order was used for each run. At trial onset, a fixation cross remained in the center of the monitor for 1 s. Target and neutral-match words were then presented (one in the left and right visual field) for 1 s. For some trials, the cross was shown in a space previously congruent with target presentation, and others, in the incongruent space. The number of congruent and incongruent trials was roughly balanced. The visual field of presentation for target and match words was randomized. After the words, a cross appeared randomly on either the left or right side, and participants had 3 s to respond to it by uncrossed left or right button press.

#### Psychophysiological measurement

Psychophysiological data were acquired with JLC physiological sensors, equipment, and software and with the data-acquisition program Snap-Master™. The physiological measures were digitized at 512 samples per second with a 31 channel analog to digital (A/D) converter with a resolution of 12 bits and an input range of −2.5 V to +2.5 V. All signals were sent to a JLC bioamplifier (Model NP-10A) and converted offline for storage and analysis.

#### Heart rate

HR is a measure of the activity of the heart and is known to increase with SNS activation, PNS withdrawal, or both (Andreassi, 2007). Sensors were placed on the left and right forearms to continuously record the ECG from which inter-beat intervals were calculated and then converted to HR in beats per minute.

#### Respiratory sinus arrhythmia

RSA indexes PNS activity (Porges, 2007) and was derived from measures of heart rate and respiration (the latter, from a respiration belt around the torso) using custom software. RSA in seconds was computed by calculating the difference in minimum heart period during inhalation and maximum heart period during exhalation twice for each respiration cycle, with the midpoint assigned as the RSA value (Grossman, 1992).

#### Skin conductance level

SCL indexes transient changes in the skin's electrical conductance. SCL is associated with changes in eccrine sweat gland activity in response to arousing stimuli and is primarily mediated by frontal, limbic, and hypothalamic pathways via the SNS (e.g., Williams et al., 2001). SCL changes are interpreted as signals of orienting, attention, and affective response (Andreassi, 2007). SCL was measured in microSiemens, using 8 mm Ag/AgCl sensors attached to the left middle and index fingers that transmitted a constant 0.5 vrms/30 Hz voltage.

### Procedures

Data reported in the present paper were collected as part of a larger IRB-approved study of survivors of IPV (D'Andea & Pole, 2012). Only the pertinent experimental procedures are included here. After providing informed consent, participants completed self-report measures. They then had sensors affixed to measure physiological activity. After a 100 s rest period during which baseline physiological data were collected, participants responded to the dot probe task, itself lasting approximately 10 min. Following completion of these and all other experimental tasks, participants were thanked for their participation, debriefed, and compensated $75 for their time.

### Data reduction

Physiological data (HR, RSA, and SCL) were aggregated across the baseline period, providing mean scores for analysis. The range of useable task reaction times (RTs) was 100–3,000 ms (Dalgleish et al., 2001), excluding 0.51% of data. In addition, 0.36% of data were excluded due to errors (e.g. indicating the probe on the incorrect side). One neutral trial was omitted entirely because RTs (*M*=943.56, *SD*=625.35) differed significantly from overall RTs excluding this trial (*M*=503.07, *SD*=140.72), *t*(20)=3.37, *p*<0.01, *d*=0.97.

### Data analysis

#### Attention bias

MacLeod et al.'s (1986) formula was applied to RT data to compute bias scores. We presented stimuli to the left and right of central fixation giving four combinations (right probe–right target [RPRT], left probe–left target [LPLT], right probe–left target [RPLT], and left probe–right target [LPRT]), and the following bias formula: [(LPRT+RPLT)−(RPRT+LPLT)]/2.^1^ Positive values are interpreted as bias toward target stimuli, and negative values, bias away from these stimuli.

#### Hypothesis testing

Repeated-measures ANOVAs were used to test for main effects of word type (neutral, positive, anxiety, or IPV-related) on reaction time and bias. Paired-samples *t*-tests were used for post-hoc tests following ANOVA to determine if emotion bias scores differed from the neutral condition. Pearson's correlations were used to determine the relationships among physiological measures, attention bias, and symptoms.

## Results

### Trauma exposure

All participants reported at least one assault by a caregiver or intimate partner. Eighteen participants (78%) reported non-sexual physical assaults, 18 (78%) reported sexual assaults; and 14 (52%) reported both; and all had interpersonal violence exposure for 1 year or more, which is one proposed chronicity cut-off for complex trauma (van der Kolk et al., 2009). Seventeen participants (74%) reported at least one non-interpersonal trauma (e.g. accident or natural disaster). All participants experienced trauma prior to adulthood; the mean earliest age for any trauma was 6.4 years (*SD*=5.9). All but one participant reported adulthood trauma. The mean age for most recent trauma was 35.5 years (*SD*=13.9). The sample reported 5–25 (*M*=11.34, *SD*=5.9) types of traumas over their lifespan.

### Self-reported psychopathology

The sample reported more severe dissociative symptoms than outpatient samples with PTSD (Carlson & Putnam, 1993) and greater PTSD symptoms than trauma-exposed college students (Ruggiero et al., 2003). All participants had PCL scores in the clinical range for PTSD. Fifteen participants (65.2%) were above clinical cut-off scores on the DES (Carlson & Putnam, 1993) and the DES-Taxon (Waller & Ross, 1997), suggesting that the majority of participants experienced significant dissociation (see Table 1).

**Table 1:** Descriptive statistics for baseline symptoms and trauma exposure assessed by self-report, reaction time and bias scores for the dot probe conditions, and baseline physiology

	M	SD	Min	Max
*Self-report*				
DES	39.91	19.37	6.79	76.79
PCL total score	35.77	12.86	14.00	58.00
PCL intrusion	10.23	5.12	3.00	20.00
PCL avoidance	14.18	5.93	3.00	26.00
PCL hyperarousal	11.36	4.23	4.00	18.00
THQ trauma type count	11.23	5.56	5.00	25.00
THQ age at earliest trauma	6.78	5.97	0.00	22.00
*Reaction time*				
Neutral	526.23	151.14	339.42	850.23
Positive	503.29	143.22	337.94	823.64
Anxiety	514.60	150.65	327.82	841.23
IPV threat	518.36	159.28	339.53	888.65
*Bias scores*				
Neutral	233.17	318.18	−116.16	1015.07
Positive	−14.20	62.96	−113.58	131.29
Anxiety	−246.89	111.22	−536.74	48.64
IPV threat	17.04	43.93	−132.83	92.36
*Physiology*				
Baseline HR	62.29	15.51	43.29	105.57
Baseline RSA	0.07	0.08	0.01	0.38
Baseline SCL	4.20	1.78	0.74	7.95

Note: PCL values are presented here as symptom intensity total scores. THQ trauma type data represent a raw count of the number of different types of traumas participants endorsed over their lifespan. Bias score and reaction time data are presented in milliseconds.

DES=dissociative experiences scale; PCL=PTSD Checklist – Civilian Version; THQ=trauma history questionnaire; IPV=interpersonal violence; HR=heart rate (BPM); RSA=respiratory sinus arrhythmia (arbitrary units); SCL=skin conductance level (microSiemens).

### Attention data

Dot probe descriptives are in Table 1. A repeated-measures ANOVA showed no significant main effect of emotion on RT (*F*[2.15, 55.96]=1.83, *p>*0.05, ${\text{η}}_{\text{p}}^{\text{2}}$=0.07). For the neutral condition, there was no difference in RT between congruent and incongruent trials. However, there was a significant main effect of trial valence on bias scores (*F*[1.313, 26.26]=26.00, *p<*0.001, ${\text{η}}_{\text{p}}^{\text{2}}$=0.565). Post-hoc *t*-tests revealed all contrasts were statistically significant (see Table 2). Bias scores for positive and anxiety words were negative, indicating a bias away from these stimuli. The IPV bias score was positive, indicating a bias toward these stimuli.

**Table 2:** Post-hoc paired-samples *t*-tests for bias scores from the 4 dot probe conditions

Comparison	*t*(df)	*p*
Neutral–positive	3.33(20)	0.003
Neutral–anxiety	6.50(19)	0.000
Neutral–IPV	2.99(20)	0.007
Positive–anxiety	8.33(25)	0.000
Positive–IPV	−2.49(26)	0.020
Anxiety–IPV	−11.19(25)	0.000

IPV=interpersonal violence.

### Are there relationships between attention bias and self-reported experiences?

Positive cue bias was not significantly correlated with self-reported symptoms or trauma history. Bias away from anxiety cues was marginally correlated with intrusion symptoms (*r*=−0.40, *p*<0.10). Anxiety bias was not correlated with other self-report variable. Bias toward IPV words was marginally negatively correlated with intrusion (*r*=−0.37, *p*<0.10) and avoidance symptoms (*r*=−0.39, *p*<0.10), and significantly negatively correlated with PTSD total score (*r=*−0.43, *p*<0.05). IPV bias was not correlated with trauma history variables (see Table 3).

**Table 3:** Zero-order correlations for study variables

	Symptoms					Trauma		Bias			Physiology		
				
	Intrus	Avoid	Hyper	Sum	DES	Age	No. of exposures	Pos	Anx	IPV	HR	RSA	SCL
Symptoms													
Intrusion	–	**0.42**	**0.60**	**0.77**	−0.01	0.20	0.20	0.01	0.40+	−0.37+	0.14	−0.39+	−0.32
Avoidance		–	**0.69**	**0.85**	0.28	−0.19	−0.19	0.22	0.21	−0.39+	0.22	**−0.50**	0.01
Hyperarousal			–	**0.88**	0.34	−0.06	−0.06	0.01	−0.07	−0.33	0.04	−0.17	−0.10
PCL Sum				–	0.13	0.10	−0.06	0.10	0.18	**−0.43**	0.18	−0.41+	−0.20
DES					–	−0.29	0.55	0.11	0.17	−0.34	−0.15	−0.11	−0.38+
Trauma													
Age, 1st trauma						–	−0.32	0.04	0.34	0.04	**0.42**	−0.30	0.20
No. of exposures							–	0.06	0.27	−0.12	**−0.44**	−0.01	−0.25
Bias													
Positive								–	0.11	0.32	**0.42**	0.08	−0.18
Anxiety									–	−0.05	−0.34	**−0.45**	**0.53**
IPV										–	0.08	0.05	−0.01

Note: Significant correlations (*p*<0.05) are bolded.

+ Indicates marginal significance (*p*<0.10). Neutral bias scores are intentionally omitted for the convenience of the reader, but nonetheless there were no significant or marginal correlations between neutral bias scores and self-report.

Intrusion=PCL intrusion total score; avoidance=PCL avoidance total score; hyperarousal=PCL hyperarousal total score; sum and PCL sum=PCL total score; DES=DES total score; age, 1st trauma=age at first reported trauma; No. of exposures=number of different types of trauma participants reported experiencing; IPV=interpersonal violence; HR=heart rate; RSA=respiratory sinus arrhythmia; SCL=skin conductance level.

### Are there relationships between attention bias and physiology?

Table 1 lists descriptives for baseline physiology. Positive cue bias scores were significantly correlated with baseline HR (*r*=0.42, *p*<0.05); positive cue bias was not correlated with RSA or SCL. Bias toward anxiety cues was negatively correlated with RSA (*r*=−0.45, *p*<0.01) and positively correlated with SCL (*r*=0.53, *p*<0.05). There was no significant correlation between IPV bias and physiology (see Table 3).

### Are there relations among symptoms, trauma exposure, and physiology?

#### Symptoms

Individuals with greater PTSD intrusion symptoms had lower RSA (*r*=−0.50, *p<*0.05); intrusion symptoms were not correlated with HR or SCL. Individuals with higher avoidance symptoms had significantly lower baseline RSA (*r*=−0.50, *p*<0.05). There was no relationship between avoidance and HR or SCL. Hyperarousal scores were not correlated with physiology. Participants with higher PCL total scores had marginally lower RSA (*r*=−0.41, *p*=0.10). There were no relationships between total PCL score and HR or SCL. DES scores were marginally negatively correlated with SCL (*r*=−0.38, *p=*0.08), but not with HR or RSA.

#### Trauma exposure

Age at earliest trauma (*r*=0.42, *p*<0.05) and number of types of lifetime traumas experienced (*r*=−0.45, *p*<0.05) were both negatively correlated with baseline HR. There were no other correlations between trauma exposure and physiology (see Table 3).

## Discussion

The present study examined attention bias and its self-report and physiological correlates in women (*n*=27) with chronic IPV exposure. Results indicated that relative to neutral cues, the sample demonstrated bias to all emotion cues (positive, anxiety, and IPV) on a dot probe task. The direction of bias differed by valence. Biases were away from positive and anxiety words, but toward IPV words. Attention bias was related to physiology and self-report symptoms, and physiological activity (RSA, HR, and SCL) differentiated symptoms and trauma exposure.

Positive biases were associated with less severe trauma exposure and higher HR. We found bias toward positive cues was associated with less severe exposure, which possibly suggests that this bias may serve a protective role. As participants who had higher HR also had increased bias to positive stimuli, the following are possible: 1) higher HR may allow participants to engage positive material, consistent with Hamann, Ely, Grafton, and Kilts (1999), who found a correlation between increased amygdala activity during encoding and better later recall of positive stimuli, and 2) individuals with both higher resting HR and less severe exposure may be less likely to view positive material as aversive whereas lower HR and more severe exposure may be associated with disengagement. Experiences of negative emotions during potentially positive events (hedonic interference) are reported in adult survivors of childhood trauma (Frewen, Dean, & Lanius, 2012).

This interpretation of increased arousal as potentially protective is mirrored in RSA and SCL findings. Decreased baseline RSA, indexing reduced PNS activity, was associated with avoidance of anxiety cues, and increased intrusion and avoidance symptoms. These findings conflict with the prior relationship found between low baseline RSA and threat bias in healthy controls (Cocia et al., 2012) and suggest that survivors of IPV with compromised emotion regulation direct attention away from general anxiety cues, perhaps because their physiology cannot sustain this distress. Hansen et al. (2003) have suggested that RSA supports attention maintenance during challenging tasks; thus, we might expect lower RSA to relate to avoidance of difficult material.

Total PTSD symptoms were correlated with IPV bias, indicating that participants with more intense symptoms showed avoidance of trauma cues. On the symptom-domain level, there were trend level negative correlations between avoidance and intrusive symptoms, and bias for IPV cues, suggesting that elevations in these symptoms were associated with avoidance of trauma cues. Avoidance and intrusive symptoms findings are consistent with prior studies (Elsesser et al., 2004; Teachman et al., 2007), suggesting some generalizability of anxiety disorder/PTSD mechanisms to chronic IPV populations. Yet as a group, participants generally directed attention away from anxiety cues but toward trauma-specific cues. Perhaps complexly traumatized individuals may manage distress through disengagement when stressors are low-grade, and are less able to when threats are more severe.

While research suggests increased subjective distress accompanies increased autonomic arousal for most individuals on the anxiety disorder spectrum, chronic PTSD appears to be one exception. McTeague et al. (2010) demonstrated that individuals with PTSD from chronic IPV exposure show high subjective distress but blunted defensive startle response to personalized threat cues. Our study shows a similar split between self-report and lab measures. Symptoms were related to IPV bias and baseline physiology (RSA), but physiology was only related to anxiety cue and not IPV bias. This divergence may suggest that among complexly traumatized individuals, level of attention for threat may mesh with physiology, but self-report may be less likely to detect alterations in either of these domains. Future studies should integrate physiology and attention with larger samples stratified by trauma history and diagnostic co-morbidity.

Increased intrusion and avoidance symptoms were related to lower baseline RSA, increased dissociative symptoms were related to marginally lower SCL, and participants with a broader and earlier trauma histories had lower HR. Our findings expand upon and clarify prior studies (e.g. Griffin et al., 1997; Hopper et al., 2006; McTeague et al., 2010; Sack et al., 2004) and further suggest that a blunted rather than elevated pattern of physiological activity accompanies elevated symptoms associated with chronic trauma exposure. Blunted HR and SCL are likely underpinned in part by low amygdala activity, itself found in individuals co-morbid for PTSD, and borderline personality disorder during painful stimulation (Kraus et al., 2009). With regards to RSA, the negative correlations between this measure and avoidance and intrusive symptoms may provide evidence of linkages between a physiological marker of behavioral flexibility and poor capacity to regulate attention to trauma material.

The present study is not without limitations. Due to the parent study design (D'Andea & Pole, 2012), there is no concurrent data from non-PTSD-affected or non-IPV-exposed controls. We do report on a number of significant findings using dimensional symptom data in our sample. In addition, due to sample size, Type II errors may have occurred. Reporting marginally significant finding was one guard against Type II error. The present study also cannot determine if the various components of dissociation (e.g. depersonalization/derealization, identity fragmentation, shifts in self state) were related to attention or physiology. Further, nearly half the sample was taking psychotropics known to influence autonomic activity (e.g. Licht, de Geus, van Dyck, & Penninx, 2010), but we could not investigate these effects due to our sample size. It could be argued that the study is limited because it did not use ideographic trauma stimuli. However, a larger proportion of IPV words relative to others were used to be more inclusive with respect to IPV experiences, and the stimuli used in the present study have yielded results in trauma-exposed samples (Pole, 2007).

This study provided evidence for relationships among physiological activity, self-reported experiences, and attention biases in women with IPV exposure. Because the sample had heightened symptom severity and a wide breadth of trauma exposure, points of convergence with and divergence from prior findings are notable. Our participants appear similar to those on the anxiety disorder spectrum (e.g. Elsesser et al., 2004; Teachman et al., 2007) in terms of the relationship between avoidance and intrusive symptoms, and threat processing. But our findings, particularly relating elements of attention and symptom distress to low rather than high physiological arousal, argue for the importance of examining attention and physiology in individuals whose posttrauma pathology is severe and exposure chronic. For example, the treatment implications of blunted physiology are a new topic (D'Andea & Pole, 2012). Overall, we are optimistic that our findings fit into a growing body of research attending to individuals with high clinical need.

## Footnotes

1 It was determined during analysis that no LPRT trials were programmed for the anxiety condition. Therefore, the following derivation of the bias formula was used to compute the bias score for this condition: bias score=[((RPLT)−(LPLT+RPRT))/2].
